# Spatial and Temporal Patterns of Prion Gene Variation Are Consistent With a Response to Chronic Wasting Disease‐Induced Selection in Wild White‐Tailed Deer

**DOI:** 10.1002/ece3.72449

**Published:** 2025-11-14

**Authors:** Christine M. Bubac, Ty Russell, Debbie McKenzie, Mark C. Ball, Margo J. Pybus, David W. Coltman, Catherine I. Cullingham

**Affiliations:** ^1^ Department of Biology Carleton University Ottawa Ontario Canada; ^2^ Department of Biological Sciences University of Alberta Edmonton Alberta Canada; ^3^ Alberta Environment and Protected Areas Fish and Wildlife Stewardship Edmonton Alberta Canada; ^4^ Biology Department Western University London Ontario Canada

**Keywords:** cervid, chronic wasting disease, prion, *PRNP*, transmissible spongiform encephalopathy, White‐tailed deer

## Abstract

Chronic wasting disease (CWD) poses a threat to cervids and is increasingly prevalent throughout North America. Prion protein gene (*PRNP*) variation may confer some degree of genetic resilience, creating an impetus to examine changes in allelic variation and to assess signatures of selection. We investigated the association between CWD and *PRNP* variation in white‐tailed deer (WTD) ( *Odocoileus virginianus* ) and mule deer (MD) ( *Odocoileus hemionus* ) sampled in Alberta, Canada between 2014 and 2017. We sequenced the *PRNP* gene of 575 WTD (67 CWD‐positives) and 660 MD (202 CWD‐positives) and detected 14 single nucleotide polymorphic loci in WTD and 8 in MD. No association was identified between the MD genetic variation and disease status. Notably, a variant at 286 was detected in WTD, resulting in an amino acid change at codon 96 (G96S). Genotype counts at this locus were significantly associated with CWD status, with the 96S allele under‐represented among CWD‐positive and over‐represented among negative individuals. For a CWD‐positive individual, the odds of being homozygous for the major allele (G96/G96) were more than sevenfold greater than being homozygous for the minor allele (96S/96S). Following additional sequencing of 1612 WTD, we examined spatial and temporal variability of this locus in association with the disease history on the landscape. Among females, the frequency of 96S varied negatively with the distance to where CWD was first detected. Additionally, the 96S allele frequency has increased over time, in line with expectations based on estimated disease selection coefficients. Our results are consistent with CWD selection pressure resulting in increasing frequency of the 96S allele in space and time, indicating it may confer some resilience and extended infection with CWD.

## Graphical abstract

In this study, we investigated the association between chronic wasting disease (CWD) and variation in the prion protein gene (PRNP) in white‐tailed deer and mule deer sampled in Alberta, Canada between 2014 and 2017. Our results are consistent with CWD selection pressure resulting in increasing frequency of the 96S allele in space and time, indicating it may confer some resilience and extended infection with CWD.

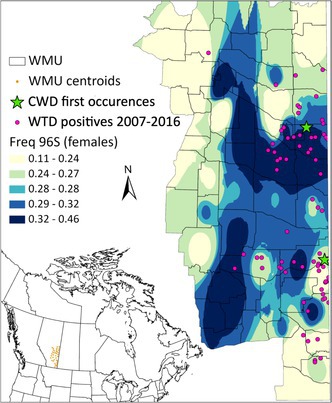

## 1 Introduction

Areas affected by chronic wasting disease (CWD) have increased in North America since the disease was first documented in wild cervids in 1981 (Spraker et al. 1997). Chronic wasting disease is a fatal prion disease, also known as a transmissible spongiform encephalopathy (TSE), characterized by an extended asymptomatic phase that is followed by the presentation of neurodegenerative conditions that eventually lead to death (Prusiner 1998). Chronic wasting disease can be transmitted when diseased individuals shed infectious prions through various biological materials including saliva, urine, feces, blood, and carcasses (Haley et al. 2011; Mathiason et al. 2006). These infectious prions can persist in the environment for multiple years (Mathiason et al. 2009). Individuals within a population could become infected through direct contact with a CWD‐infected animal or indirectly via environmental contamination of shed prions. Currently, CWD has been identified in several cervid species including mule deer (MD) ( *Odocoileus hemionus* ), white‐tailed deer (WTD) ( *Odocoileus virginianus* ), elk ( *Cervus canadensis* ), and moose ( *Alces alces* ) in North America, and red deer ( *C. elaphus* ), moose, and caribou ( *Rangifer tarandus* ) in Europe (Baeten et al. 2007; Benestad et al. 2016; Williams et al. 2002).

While research is underway to develop vaccines and non‐invasive assays for pre‐symptomatic diagnoses (Goñi et al. 2015; John et al. 2013; Napper and Schatzl 2023a), there is currently no treatment or cure for CWD. Variation in the prion protein gene (*PRNP*) may, however, provide insight into CWD susceptibility and rate of disease progression. *PRNP* encodes the amino acid sequence of the cellular prion protein (Prusiner 1998). All mammals have cellular prion proteins; though, the introduction of pathogenic prion particles causes chain reactions of misfolding in the cellular form, leading to pathogenic prion aggregations, accumulations, and fatal TSEs (Aguzzi et al. 2008). As such, *PRNP* allelic variation that alters the amino acid sequence may affect the propensity for misfolding, thereby influencing CWD susceptibility and progression (Angers et al. 2014). *PRNP* genotype in WTD, for instance, has been linked to lower CWD prevalence in deer having the 96S allele in comparison to their 96GG counterparts (Johnson et al. 2003, 2006; Robinson et al. 2012; Wilson et al. 2009). Though rare in wild WTD populations (Robinson et al. 2012; Arifin et al. 2021), a Q95H polymorphism may similarly provide protection by reducing CWD susceptibility (e.g., Johnson et al. 2006 and Ishida et al. 2020). In MD, Wilson et al. (2009) found a CWD association at codon 20, where individuals homozygous for the major allele (aspartic acid) were less likely to test positive for CWD than expected based on the allele frequency in the population. Further, variation at codon 225 was associated with disease status in MD of Wyoming and Colorado, USA (Jewell et al. 2005; LaCava et al. 2021). Among elk, O'Rourke et al. (2007) found individuals homozygous for the minor allele (132LL) showed reduced CWD progression rates with extended incubation periods when compared with elk homozygous for the major allele (132MM). Finally, in caribou, an amino acid change at position 138 from serine to asparagine (138 N) conferred resistance in a CWD challenge experiment (Mitchell et al. 2012).

To date, there have not been any *PRNP* allele variants linked to complete genetic resistance to CWD; however, even a small increase in resilience or slowed disease progression for particular alleles may confer herd advantage. This is important given the negative impacts and significant regional declines that endemic CWD is having on cervid populations throughout North America (DeVivo et al. 2017; Edmunds et al. 2016). When selection pressure is strong, as may be the case with disease in naïve and/or densely populated areas, shifts in allelic distribution patterns across the landscape may emerge, even over relatively short timescales (Gallana et al. 2013). Enhancing our understanding of factors associated with CWD epidemiology is essential given the substantial economic costs (Chiavacci 2022; Napper and Schatzl 2023b), cultural effects (Vaske 2010; Parlee et al. 2021), zoonotic potential (Trout et al. 2024), and evolutionary implications of CWD on cervids as well as their management.

Mule deer currently have the highest rate of CWD infection in Canada (Smolko et al. 2021), and are widely distributed in western North America (Innes 2013), overlapping with other common ungulates including WTD. Mule deer and WTD exhibit high mobility and have well‐described social structures (Hirth 1977; Brunjes et al. 2006; Latch et al. 2014; Airst and Lingle 2019). As seasonal migrants, deer concentrate in areas of less snow accumulation during the winter months before dispersing and moving to more suitable birthing and foraging grounds in spring/summer. Males are the dispersive sex and compete for access to mating opportunities during rut but later form small bachelor groups following each breeding season. In many CWD‐affected areas, males are up to three times more likely to be infected with CWD than females in a population (Rogers et al. 2022; Smolko et al. 2021; LaCava et al. 2021), though the exact source of this sex effect is unknown and requires further investigation. Behavior, social structure, and demographics of deer may therefore influence the prevalence, spread, and rate of CWD infection within and across populations. In western Canada, for instance, CWD‐infected deer were more closely related to each other and thus likely shared a common space and were in closer contact compared to noninfected matched pairs (Cullingham, Nakada, et al. 2011). In addition to behavior, deer inhabiting environments heavily contaminated with infectious prions may be at higher risk of exposure and subsequent CWD infectivity (Almberg et al. 2011). This indirect mode of transmission has been suggestively linked with a significant increase in CWD cases in areas marked by persistent CWD infection for 10 years or longer (Smolko et al. 2021).

In Canada, CWD was first detected in farmed elk in Saskatchewan in 1996 (Kahn et al. 2004). It then spilled over into wild deer, spreading into other provinces including Alberta (detected in 2005) (Smolko et al. 2021), Manitoba (detected in 2021) (Thompson et al. 2023), and British Columbia (detected in 2024) (Government of British Columbia 2025). This spread creates an impetus to understand the spatial and temporal dynamics of CWD across the landscape. Previous research identified genetic variants in the *PRNP* gene that were associated with CWD status in both MD and WTD (Jewell et al. 2005; Johnson et al. 2003, 2006; Wilson et al. 2009; LaCava et al. 2021). In these studies, CWD‐positive individuals with the resilient genotype were identified, suggesting that *PRNP* genotype does not confer complete disease resistance but rather slows disease progression by, for instance, increasing incubation time (Race et al. 2011). Thus, we would expect that the more resilient associated allele would increase over time and space in association with the disease history on the landscape. To investigate this, we examined the *PRNP* allele frequency distribution in an outbreak region within Canada using high‐throughput DNA sequencing data and longitudinal disease prevalence data (2005–2017) for MD and WTD. Our objectives were to: (a) identify additional *PRNP* genetic variants, (b) determine whether variants are associated with CWD status, (c) for those that are associated with CWD status, assess whether there is a shift in variant frequency over time, and (d) characterize the spatial distribution of CWD‐associated alleles. While these populations are weakly genetically structured (Cullingham, Nakada, et al. 2011; Cullingham, Merrill, et al. 2011), selection pressure can result in spatial variation in allele frequencies (Robinson et al. 2012), such that resilient alleles will be more frequent in areas where CWD has been more prevalent.

## 2 Methods

### 2.1 Sampling

**FIGURE 1:**
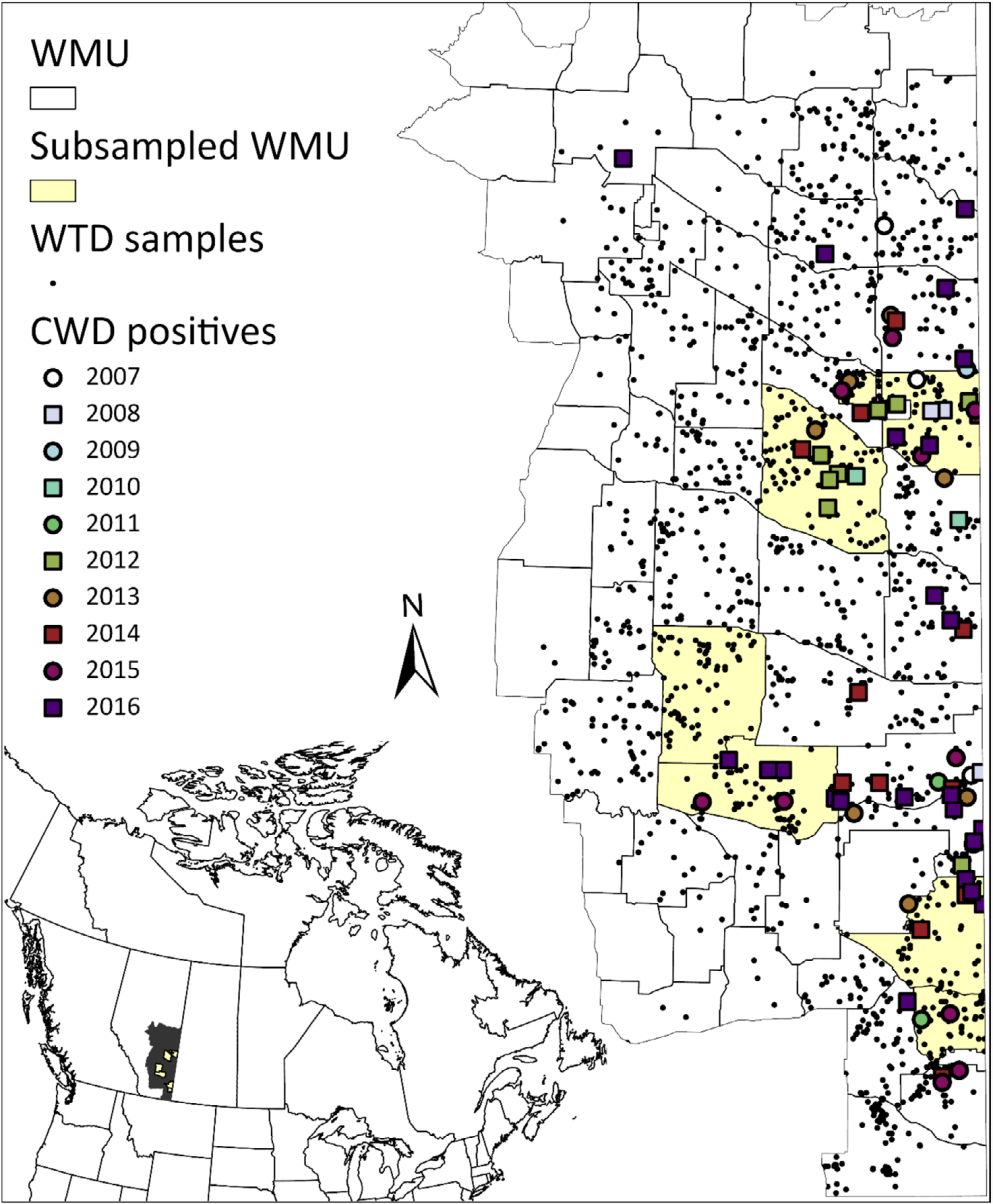
White‐tailed deer (WTD) sampling within wildlife management units (WMUs; 2014–2017), as well as of chronic wasting disease (CWD) occurrence in WTD, in Alberta (Canada) from 2007 to 2016. The seven wildlife management units (WMUs) used for the subsampling are highlighted in yellow.

Mule deer and WTD samples used for this study were selected from the Alberta Environment and Protected Areas CWD surveillance program from the years 2014 to 2017 (Figure 1). Each year, hunters submit heads of harvested deer for CWD testing and a small portion of the animal's ear was obtained for genetic analysis. The surveillance program at that time was focused on the southeastern region of Alberta. Deer were tested for CWD using Bio‐Rad TeSeE test assays (Bio‐Rad, Hercules, California, USA) by the TSE testing laboratory with the Alberta Department of Agriculture and Irrigation. Standard immunohistochemistry was also performed for confirmation by the Canadian Food Inspection Agency in Ottawa, Government of Canada (see Cullingham, Nakada, et al. 2011).

We first selected samples for *PRNP* sequencing of both species to estimate the number of samples required to ensure we were capturing allelic diversity within the scale of a wildlife management unit (WMU) (Figure S1), and to assess the relationship between *PRNP* polymorphisms and CWD status. We selected seven WMUs and randomly selected a minimum of 40 individuals of each species to sequence within each unit (Table 1) and all CWD‐positive samples that were available. For the second set of samples, we focused on WTD and aimed to sample a minimum of 10 males and 10 females in each available WMU.

**TABLE 1:** Number of individual deer (MD = mule deer, WTD = white‐tailed deer) sampled in each wildlife management unit (WMU) to determine the optimal sampling size to capture frequencies of genetic variants within the *PRNP* gene. For the location of each WMU, please refer to Figure S1.

WMU	MD (*N*)	WTD (*N*)
119	92	65
148	41	79
152	39	103
160	113	97
202	45	54
234	77	86
728	259	48
Positives^a^	202	43
Total	660	575

^a^ These are CWD‐positive individuals that were sampled in other wildlife management units.

### 2.2 DNA Extraction and *PRNP* Sequencing

We extracted DNA from ear tissue samples using Qiagen DNeasy Blood and Tissue Kits (Qiagen, Mississauga, ON) following the manufacturer's protocol but eliminating the second elution step. Extracts were quantified using a NanoDrop 2000 (Thermo Fisher Scientific, Waltham, MA). Amplification and high‐throughput sequencing of the *PRNP* gene on an Illumina MiSeq are described by Cullingham et al. (2020).

### 2.3 Sequence Assembly and Polymorphism Analysis

All MiSeq sequence data were uploaded to BaseSpace (Illumina). Following indexing of all samples, run data were downloaded and processed in Geneious Prime (Biomatters, Aukland, New Zealand). Once we set paired reads, reads were trimmed using BBDuk2 (https://sourceforge.net/projects/bbmap/, accessed 02‐08‐2021) with the following parameters: qtrim = rl, trimq = 20, minlength = 100, ordered = t, and qin = 33. Each individual was assembled to cervid *PRNP* [Accession: AY228473 (Jewell et al. 2005)] using the Geneious alignment algorithm and parameters including minimum quality = 30, word length = 12, and index word length = 11. The consensus sequence was saved using the “highest quality threshold” at 75%. Consensus sequences were exported as an aligned FASTA file and genotypes were called using *SNP‐sites* (Page et al. 2016).

### 2.4 Sampling Optimization

To optimize sample size to capture genetic diversity within a WMU, we estimated rarefaction curves for both species at the level of the WMU using seven different units with dense sampling (Figure 1). We developed a script in R v. 4.0.5 (R Core Team 2021) to subsample individual *PRNP* putative haplotypes at sizes of 2, 5, 10, 15, 20, 25, 30, 35, and 40 for 1000 sets each, and estimated heterozygosity using the package *gstudio* (Dyer 2021). We then estimated the average heterozygosity across each sample size and examined the rarefaction curve to select an optimal sampling size per WMU.

### 2.5 Disease Association

We used an allelic exact test in R (Guedj et al. 2006) to determine if there was a significant association between genotype and CWD status. We did this for both MD and WTD using the individuals genotyped for the sample optimization and all CWD‐positive samples [MD: *N* = 660 (202 positive); WTD: *N* = 557 (67 positive)]. If we identified an association between genetic variation and disease status for either species, we sequenced additional samples for that species across available WMUs (10 females and 10 males per unit). Using these data, we estimated odds ratios for loci associated with disease (https://www.genecalculators.net/associatorrr‐cc.html, accessed Jan 2025) and examined the temporal and spatial patterns of these loci (see below).

To further assess the sources of variation on CWD status, generalized linear models were developed using the R package *lme4* (Bates et al. 2015). Sex and genotype were included as fixed effects, and WMU was included as a random effect. An interaction term between sex and genotype was included to test for potential interactive effects. Sex and genotype were included and interpreted as independent main effects. Both additive and dominant genetic models were tested using a logit link function with a binomial distribution. For the additive model, the minor allele homozygote was coded 0, the heterozygote was coded 1, and the major allele homozygote was coded 2. For the dominant model, the minor allele homozygote was again coded 0, while the heterozygote and the major allele homozygote were coded as 1.

### 2.6 Examining Temporal and Spatial Variations in Disease‐Associated Variants

To determine whether disease‐associated alleles are more frequent in regions with a longer history of CWD, we examined the relationship between allele frequency and the number of years since CWD was first detected in each WMU. We examined this relationship for all samples as well as for males and females separately. As females show more spatial autocorrelation than males (Cullingham, Merrill, et al. 2011), we hypothesized that females would more likely show the expected pattern than males. We modeled the relationship using a beta regression. The predictor variables we considered were years since CWD was detected in the WMU (counting back from 2017 to represent an approximation of time since units have been exposed to CWD as the disease may have occurred in deer prior to detection) and distance to the two primary areas where CWD was first detected (Figures 1 and S2). Genotyping sample size was used as a weight. Modeling was completed in R, using the package *betareg* (Cribari‐Neto and Zeileis 2010). We developed an interpolated surface for the frequency of the disease‐associated allele in females across the sampled WMUs and extrapolated to units neighboring to the west and north of sampled locations. Frequency data was plotted based on the centroids of the WMUs and the frequency of the disease‐associated allele was interpolated/extrapolated using the inverse‐distance weighting function implemented in ArcGIS Pro 2.7.2 (ESRI). For the interpolation, we selected a variable search radius with a neighborhood size of 6 and a power of 2.

Selection coefficients have previously been estimated for locus G96S under four CWD selection scenarios (Haworth et al. 2021; Robinson et al. 2012). Using those coefficients, we estimated the expected allele frequency in our current dataset for G96S using data from 2005 for this population (Wilson et al. 2009). We assumed 2 years per generation (De Young et al. 2003; Kessler and Shafer 2024), using two different models of gene action: dominant and additive. We modeled this using the allele frequency from all deer genotyped in Alberta and for only WMU 151, which was sampled well by Wilson et al. (2009) and in our dataset. The equations are from Hedrick (2000):

$$q_{1}=\frac{q_{0}\left(1-sq_{0}\right)}{1-sq_{0}^{2}}\left(\text{Equation}\;3.6a\right)$$

$$\;q_{1}=\frac{q_{0}\left(1-\frac{s}{2}\left(1+q_{0}\right)\right)}{1-sq_{0}}\;\left(\text{Equation}\;3.7a\right)$$

## 3 Results

### 3.1 *PRNP* Genotyping Data

After quality filtering of individual genotype data, we obtained *PRNP* sequences for 575 WTD (67 CWD positive) and 660 MD (202 CWD positive) to assess minimum sample sizes needed to fully survey *PRNP* genetic diversity (Figures 1 and S2). We identified 14 single nucleotide polymorphism (SNP) loci in WTD and eight in MD (Table 2). Two SNPs identified in MD (413 and 417) have been identified as part of a pseudogene (nucleotides 412 and 418 in Brayton et al. 2004). For these SNPs, we did not observe any individuals homozygous for the minor allele and, of the proportion of reads, the median for the minor allele was ~29% for both loci (Figure S3). There were few individuals (*N* = 4) for whom locus 417 was heterozygous (average proportion of reads with the minor allele was 47%) and 413 was not. Four additional loci were significantly out of HWE: one locus had a large number of heterozygotes, but no minor homozygotes (437), while another had three alleles, with only the major allele being found as a homozygote (451), and finally two were heterozygous (468 and 606), suggesting fixed differences between *PRNP* and the pseudogene. On the basis of the subsampling of individuals across seven WMUs, a sample size of 15–20 individuals per WMU was sufficient to capture genetic diversity (Figure S4).

**TABLE 2:** Per locus information calculated for mule deer (*N* = 672) and white‐tailed deer (*N* = 575) in the CWD endemic region of Alberta. The PrP amino acid associated with DNA polymorphisms is indicated (A.A. change). Exact *p*‐values for Hardy–Weinberg equilibrium (HWE (*p*)) were calculated in the R package “*pegas*” (Paradis 2010) using 1000 permutations, while minor allele frequency (MAF) was estimated in GenAlEx 6.5 (Peakall and Smouse 2006, 2012), and observed (*H*_O_), and expected heterozygosity (*H*_E_) were estimated using the R package “*hierfstat*” (Goudet 2005; Goudet et al. 2022). Variants suspected to be associated with the pseudogene are bolded and the exact test was not performed as the actual genotype could not be confirmed at those loci.

Nucleotide	A.A change	HWE (*p*)	MAF	H_O_	H_E_	Exact test (*p*)
*Mule deer*						
59	D20G	1.000	0.08	0.14	0.14	0.427
393		1.000	0.08	0.15	0.15	0.067
**413**		< 0.001	0.062	0.124	0.116	
**417**		< 0.001	0.083	0.118	0.153	
**437**		< 0.001	0.15	0.29	0.25	
**451**		< 0.001	0.05	0.25	0.22	
**468**		< 0.001	0.50	1.00	0.50	
**606**		< 0.001	0.50	0.99	0.50	
*White‐tailed deer*						
60		1.000	0.04	0.07	0.07	0.638
153		0.035	0.05	0.09	0.10	1.000
243		1.000	0.02	0.05	0.05	0.761
286	G96S	0.366	0.27	0.32	0.31	< 0.001
324		0.001	0.06	0.11	0.13	0.198
347	A116G	1.000	0.05	0.08	0.08	0.671
378		1.000	0.00	0.01	0.01	0.392
437		< 0.001	0.21	0.40	0.32	0.064
438		0.246	0.08	0.13	0.15	0.871
451		1.000	0.01	0.02	0.02	0151
555		0.750	0.25	0.35	0.34	0.071
676		1.000	0.01	0.01	0.01	0.249
689	Q230L	1.000	0.01	0.02	0.02	1.000

### 3.2 Disease Association

**FIGURE 2:**
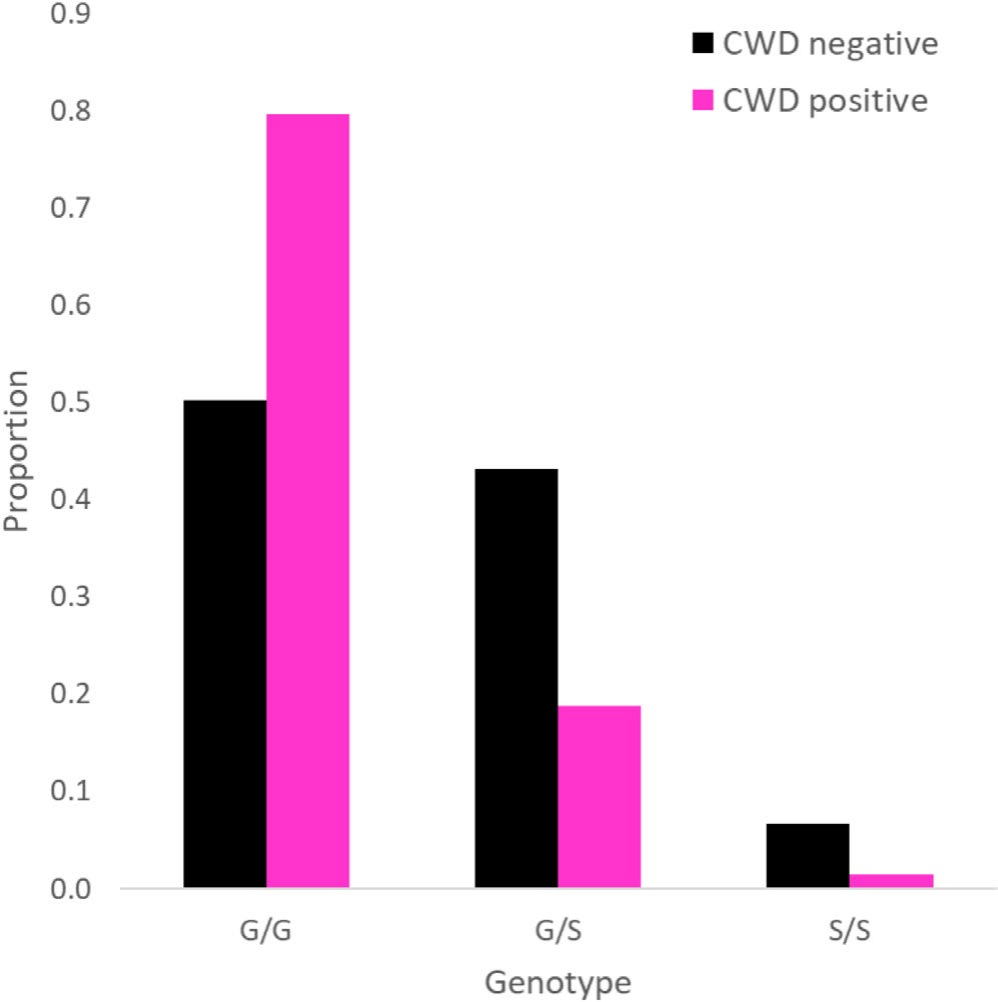
Genotype proportions for the G96S *PRNP* locus separated into CWD‐negative (black) and ‐positive (pink) individuals for white‐tailed deer in Alberta (Canada) sampled from 2014 to 2017.

Using allelic exact tests, only the variant at nucleotide 286 had a significantly different distribution of genotypes between positive and negative WTD. This variant results in a change in the prion protein at amino acid 96 from glycine (G) to serine (S) (hereafter referred to as G96S). In testing for association with disease status at WTD loci, we found G96S was significantly associated with disease status based on an allelic exact test (*p* = 4.99 × 10^−6^), with the minor allele (96S) under‐represented among CWD positive individuals and over‐represented among negative individuals (Table 3, Figure 2). The odds ratio for heterozygotes (G96/96S) at this locus is 2.09 (CI: 0.27–16.53), while that for the homozygous major allele (G96/G96) is 7.75 (CI: 1.04–57.95), indicating an increased risk of disease for individuals with those genotypes over the homozygous genotype (96S/96S). No significant association of disease and genotype from allelic exact tests was found at other loci for WTD and MD.

**TABLE 3:** Genotypes for individual white‐tailed deer, by sex, at codon 96 used to assess relationships between genotype and chronic wasting disease (CWD) status. Number of deer that tested positive for CWD are provided in parentheses.

	96S/96S (CWD +)	G96/96S (CWD +)	G96/G96 (CWD +)
Male	81 (1)	489 (12)	594 (47)
Female	57 (0)	410 (1)	450 (8)
Unknown	0 (0)	0 (0)	1 (0)
Total	138 (1)	899 (13)	1045 (55)

When we examined the relationship between genotype at codon 96 and CWD status, the interaction between sex and genotype was not significant. WMU was similarly not informative and removed from models. Genotype was an important predictor of CWD status; however, it remains unclear whether the relationship here is additive (*β* = −1.215, SE = 0.281, *p* < 0.005) or dominant (*β* = −0.675, SE = 0.152, *p* < 0.005) as both models were supported (Table S1). Sex was also a significant source of variation (*β* = 1.64, SE = 0.3611, *p* < 0.005), wherein more males were associated with CWD than females.

### 3.3 Examining Temporal and Spatial Variations in Disease‐Associated Variants

**FIGURE 3:**
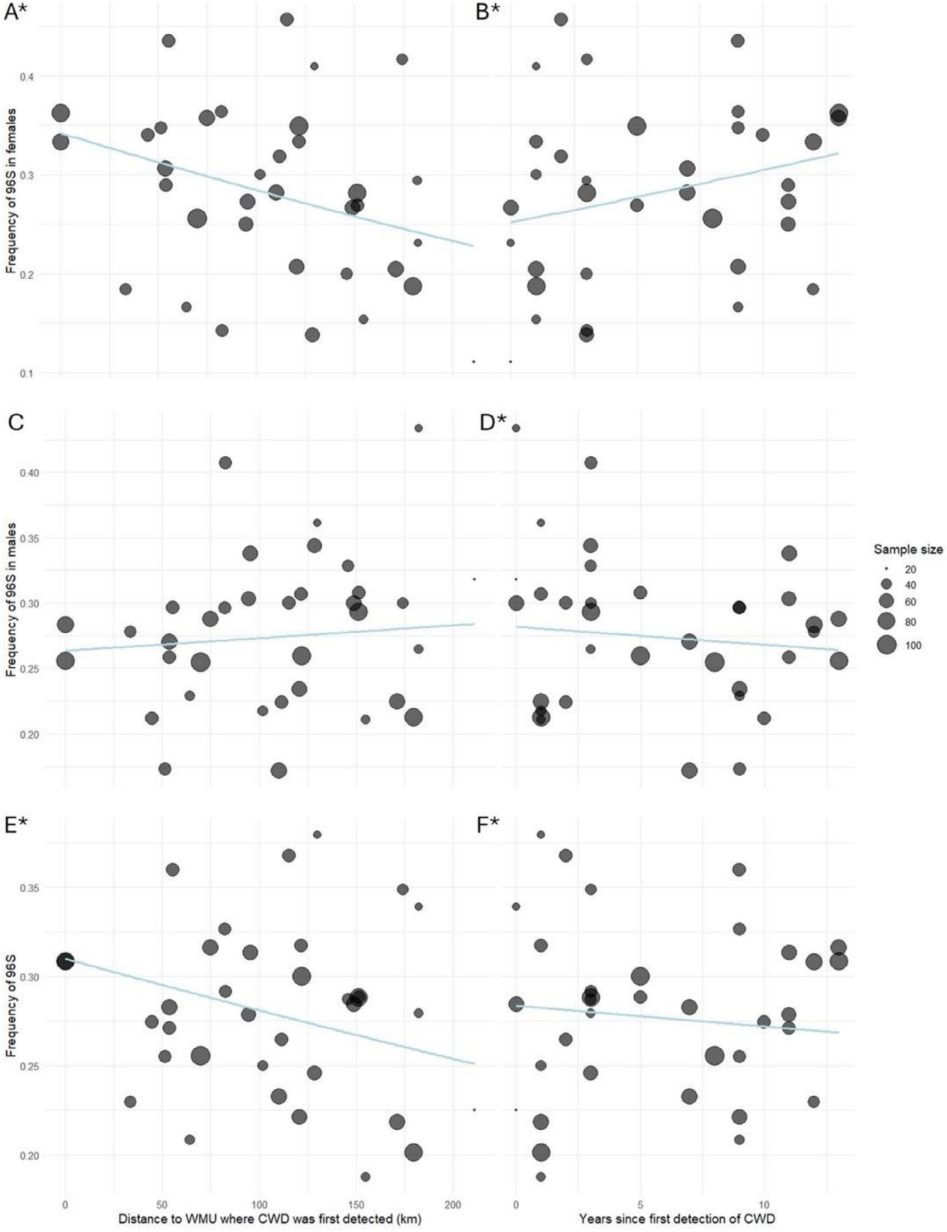
Frequency of the 96S allele estimated for 35 wildlife management units (WMUs). Panels A, C, and E show trends for the 96S allele frequency among females, males, and all deer (both sexes), respectively, versus the smallest distance of the WMU from the two regions where CWD was first detected in kilometers (km). Panels B, D, and F show trends for the 96S allele frequency among females, males, and all deer, respectively, versus the number of years since CWD was first detected (zero years are WMUs where CWD had not been detected as of 2017).

**FIGURE 4:**
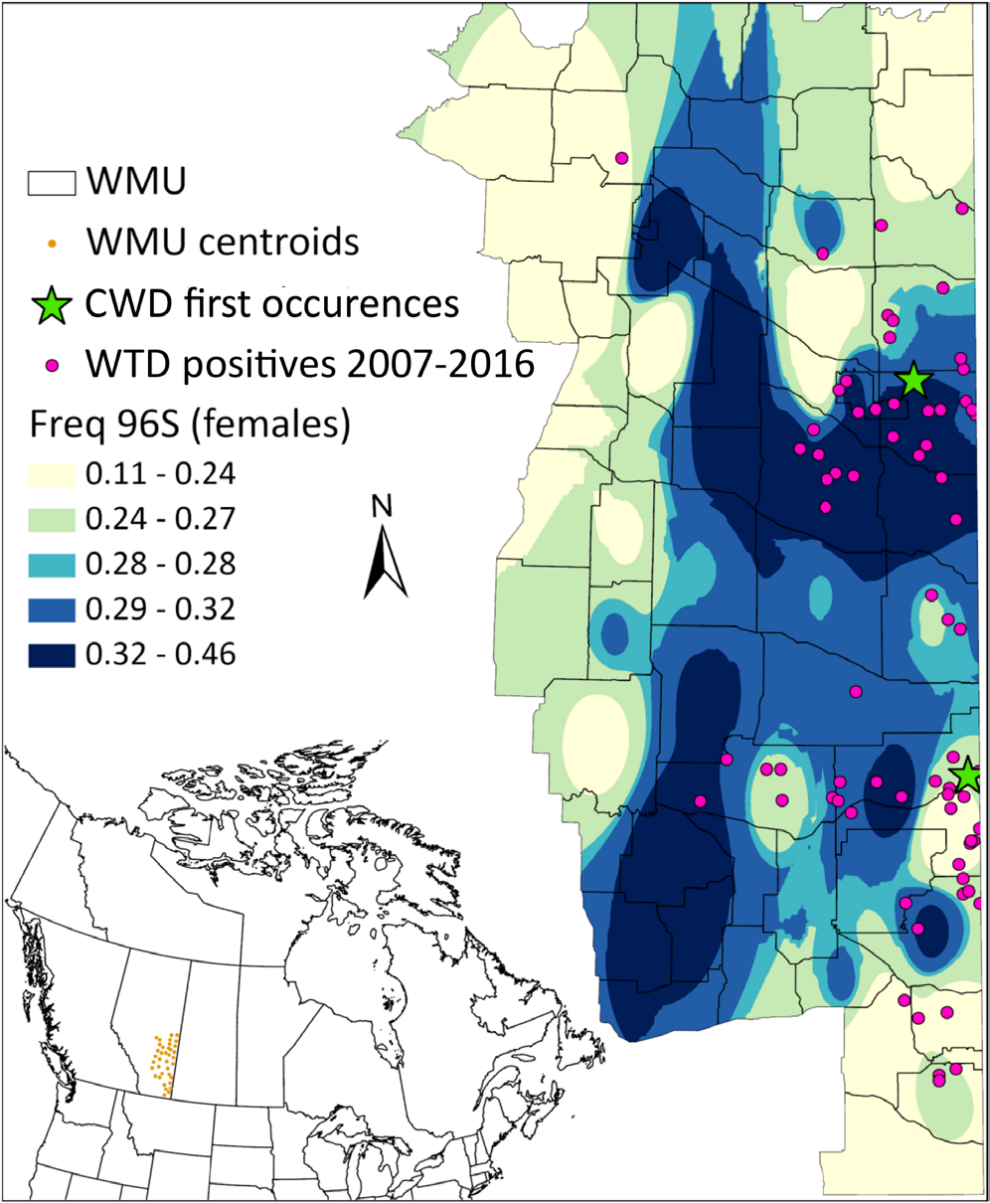
Interpolated surface map of the 96S allele frequency in female white‐tailed deer across wildlife management units (WMUs) in Alberta, Canada. Colors represent the allele frequency gradient from low (yellow) to high values (dark blue). Interpolation was performed to estimate allele frequencies at unsampled locations.

To investigate the relationship between G96S and CWD status in WTD, we sequenced an additional 1612 individuals (2 CWD positives). We combined these data with 539 of the 575 samples sequenced for the subsampling experiment, yielding a total of 2151 genotyped individuals (69 CWD positives). In total, 59 WMUs were sampled; however, 22 WMUs had fewer than 15 samples each. We therefore modeled the relationship using 35 WMUs with a total of 2012 individuals (35 CWD positives). The frequency of the minor allele, 96S, associated with CWD resilience, ranged from 0.167 to 0.379 using only CWD negative individuals. Both the time since CWD was first detected in a WMU and the distance to the locations where CWD was first detected were significant variables in explaining the frequency of 96S across the WMUs (Table 4 and Figure 3). When divided into female and male groups, 96S was more variable across the WMUs in females (0.111–0.457) than in males (0.172–0.433). We found a significant association between female allele frequency for 96S and distance to the locations where CWD was first detected (Table 4 and Figure 3), but we did not find any significant association with year. We found a significant association with male 96S frequency and the time since CWD was first detected, though the relationship was weak (Table 4 and Figure 3). The spatial distribution of the 96S allele in females was not homogeneous across the sampled landscape (Figure 4). WMUs with CWD positive cases were more likely to have higher 96S allele frequency values. WMUs west and south of the CWD endemic regions had lower 96S allele frequencies. A weak but positive correlation (*r* = 0.495) existed between the number of CWD positive cases and the number of samples used from a given WMU.

**TABLE 4:** Model outputs examining the relationship between the allele frequency of 96S sampled from white‐tailed deer in 35 wildlife management units (WMU) in Alberta and the distance to the WMU where CWD was first detected in Alberta (“Dist151‐234”) and the years since CWD was present in that WMU (CWDPresent). Three separate models were produced, one using all deer “All deer”, one with only females (“Females”), and one with only males (“Males”). Modeling was completed using beta regression in the R‐package *betareg*. Significant relationships are in bold.

	Estimate	Standard error	*z*	*p*
*All deer*				
Intercept	−0.7715	0.0274	−28.118	**< 0.001**
Dist151_234	−0.0014	0.0002	−8.683	**< 0.001**
CWDPresent	−0.0058	0.0019	−2.992	**0.003**
*Females*				
Intercept	−0.6226	0.0482	−12.928	**< 0.001**
Dist151_234	−0.0028	0.0003	−10.119	**< 0.001**
CWDPresent	−0.0024	0.0034	−0.688	0.491
*Males*				
Intercept	−0.9489	0.0337	−28.182	**< 0.001**
Dist151_234	0.0001	0.0002	0.420	0.674
CWDPresent	−0.0060	0.0024	−2.529	**0.011**

**FIGURE 5:**
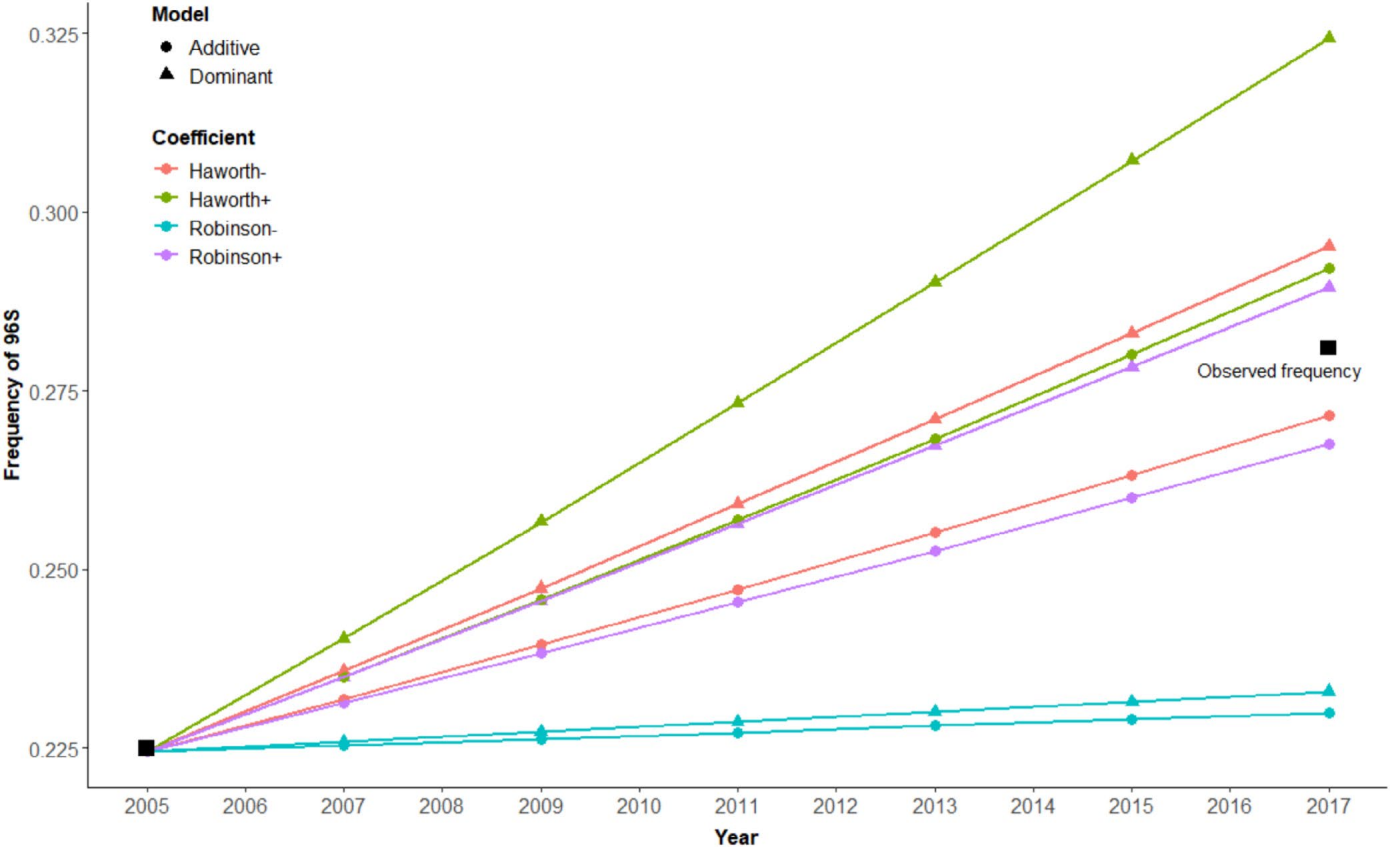
Predicted change over time in the allele frequency for 96S in white‐tailed deer based on two models of gene action (“Additive” and “Dominant”), and four estimates of selection coefficients calculated in Haworth et al. (2021), and Robinson et al. (2012). The negative “−” and plus “+” signs denote the upper and lower estimates, respectively. Observed values are denoted by black squares – the starting allele frequency for this allele was obtained from Wilson et al. (2009), and the value for 2017 was estimated from 854 individuals we sampled in 2017.

Using four selection coefficients (0.0103, 0.08, 0.074, 0.11) estimated under different scenarios (Haworth et al. 2021; Robinson et al. 2012) and two models of gene action (dominant and additive) for codon 96, we estimated the expected allele frequency over six generations based on the frequency of the 96S allele in 2005 in Alberta (0.225, *N* = 98) and in WMU 151 (0.197, *N* = 33; data from Wilson et al. 2009). The expected allele frequency in Alberta in 2017 ranged from 0.230 to 0.292 depending on the model of gene action and the coefficient used. The calculated allele frequency using 854 individuals harvested in 2017 was 0.281, falling within predictions (Figure 5), and for WMU 151 it is 0.297 (*N* = 37) which falls just above predictions (Figure S5).

## 4 Discussion

Chronic wasting disease poses a significant threat to wild cervid populations through its high transmissibility, unknown evolutionary potential, prolonged incubation periods, and certain lethality (Napper and Schatzl 2023a). Investigations into the genetic architecture of CWD in *Odocoileus* spp. may shed light on patterns of infection rates in wild cervid populations over time. Here, we contribute to a growing body of literature examining the mechanisms of CWD epidemiology by describing variation within *PRNP* in WTD and MD and reporting on the genetic association with CWD infection status, as well as the temporal and spatial variation of *PRNP* in association with disease history across the landscape.

More *PRNP* variation was observed within WTD sampled than MD, a finding similar to Wilson et al. (2009) in this region. Genome‐wide comparisons of genetic diversity between MD and WTD also found that WTD are more variable (Kessler et al. 2023). Despite sequencing a large number of samples, we did not detect any *PRNP* polymorphisms that have not been previously identified. We did not detect individuals in our WTD sample from Alberta with the 95H polymorphism—a finding not unexpected given limited variation at locus Q95H in wild deer (Robinson et al. 2012; Arifin et al. 2021). In MD, we detected variants associated with the processed pseudogene—the presence of which has not yet been linked with CWD susceptibility in deer but confounds our ability to assess *PRNP* heterogeneity accurately (Brayton et al. 2004; O'Rourke et al. 2004). Previous research conducted in MD of Wyoming and Colorado has identified variation at codon 225 linked with slowed CWD progression (e.g., Jewell et al. 2005; Wolfe et al. 2014; LaCava et al. 2021). In their analysis of spatio‐temporal patterns of codon 225, for instance, LaCava et al. (2021) found that MD possessing the 225F allele were less likely to test positive for CWD. In the present study, however, variation at codon 225 in MD was not detected. In assessing association between genetic heterogeneity and CWD incidence involving MD from Western Canada, Wilson et al. (2009) also reported no observation of variation at codon 225. The reason for this apparent lack of variation is unclear.

In WTD, SNP G96S was significantly associated with disease status. For a CWD‐positive individual, the odds of being homozygous for the major allele (G96/G96) were more than 7× greater than being homozygous for the minor allele (96S/96S). In addition, a modest reduction in the prevalence of CWD infection was observed among heterozygotes at this locus, suggesting that a single copy of the 96S allele may confer some limited resilience to infection, similar to that shown by others (e.g., Wilson et al. 2009; Robinson et al. 2012; Pilot et al. 2025). However, both additive and dominant models of gene action were supported. Although, the additive model did yield a slightly better fit to the data, and our observed change in allele frequency over time was closer to that predicted by the additive model under different selection coefficients (Figure 5). Phenotypic data from Miller et al. (2012) found that following experimental inoculation, G96/96S heterozygotes first tested positive around the same time as G96 homozygotes but survived longer, while 96S homozygotes tested positive later and survived the longest among individuals of any genotype. Cumulatively these data support an additive model, but our data involve a small number of CWD cases and additional cases would provide more power to confirm this.

In assessing the spatial and temporal trends in the G96S locus, we found multiple lines of evidence that suggest a response to selection in WTD. Spatially, we found the resilience‐conferring allele (96S) varied negatively with distance to CWD initial occurrence in females. While local female minor allele frequency (MAF) was higher in WMUs that were close to sites where CWD was first confirmed in Alberta (Figures 3 and 4), we did not find an association with male MAF (Figure 3). While sex differences in the effects may be partly due to a lack of power, several other factors may be dampening localized selection signals in male deer. For instance, selection may not be intense enough to produce noticeable effects; infected individuals often survive to reproductive age before succumbing to the disease. The absence of barriers to movement in Alberta allows WTD to disperse long distances, curbing the formation of population structure. Further, limited population structure in the prairies allows for migration from less infected areas, potentially acting as a dilution effect on selection (Munroe et al. 2015). Given their philopatric nature, however, females demonstrate stronger population structure (Cullingham, Merrill, et al. 2011), which may explain the underlying differences between allele frequency patterns in males and females as may harvest mortality. In some locations, harvest mortality is greater in antlered deer than in antlerless deer (e.g., Wasserberg et al. 2009), and males may be less likely to succumb to CWD due to this heightened harvest mortality pressure (Robinson et al. 2012). Modeling of allele frequency changes in the CWD system has demonstrated that harvest will dampen the selection pressure as disease resistance becomes less necessary to fitness (Ketz et al. 2022). Lastly, known incidence of CWD in hunter‐harvested WTD is low; therefore, selection pressure will be lower, resulting in smaller allele frequency changes over time (Ketz et al. 2022). Yet, there is undoubtedly selection in free‐ranging cervid populations we do not observe if CWD‐infected individuals do not survive to be harvested.

In experimental challenges, examination of the relationship between CWD status and codon 96 revealed that the 96S allele increased the length of the incubation period and was associated with a delay in disease onset but did not confer complete disease resistance (Race et al. 2011). Thus, it would be predicted that, upon first occurrence of the disease in a population, CWD infections would primarily occur in G96/G96 animals, and over time the relationship between genotype and CWD status would reach an equilibrium, where the proportion of animals with the 96S/96S genotype would be equivalent between CWD‐negative and CWD‐positive animals. After 15 years of CWD presence in Alberta, the association between disease status and the 96S allele has remained indicating that it could confer reduced susceptibility to infection. In support of our data, multiple studies of WTD have similarly identified relationships with the 96S allele and CWD in areas with a longer disease history (Chafin et al. 2020; Robinson et al. 2012; Johnson et al. 2006). If the locus did not confer an advantage, we would not expect to see a change in frequency over time. However, we observed the frequency of the resilient associated allele has increased over time (Figure 5). The increase in frequency is in line with predicted changes based on both additive and dominant gene models of action for different estimates of selection (estimated in: Haworth et al. 2021; Robinson et al. 2012).

The distribution of the 96S allele found among WTD females suggests that it varies spatially across the landscape, but regions with a longer history of CWD tended to have a higher frequency of the allele. Many of the WMUs to the west and south of the endemic area had a much lower frequency of the 96S allele. As such, deer populations in these units may be at somewhat greater risk of the disease. In an attempt to manage CWD, selective breeding is now occurring in farmed herds of WTD, with the goal of increasing the number of deer in captivity with alleles conferring some resilience to CWD (e.g., the 96S and 95H variants) (Haley et al. 2021). Nevertheless, there is no known complete resistance to CWD and individuals possessing more resilient alleles still acquire the disease. The spatial variation of 96S varies considerably across surveyed locations in North America (Chafin et al. 2020; Zink et al. 2020; Haworth et al. 2021), suggesting multiple factors may affect its frequency and that fitness tradeoffs (e.g., fawn mortality) in individuals having one or two copies of the allele may exist (Sawalha et al. 2010; Robinson et al. 2012; Wolfe et al. 2014). The factors contributing to the variation in the 96S allele where CWD is not an immediate threat deserve further investigation.

In the future, the 96S allele should increase in frequency in our study area as in Monello et al. (2017), who found evidence of selection for a resistant allele (132L) among North American elk (*Cervus elaphus nelsoni*) populations with 30–50 years of exposure to CWD. Hoar et al. (2024) found that an increase in CWD prevalence was associated with increased odds that an elk would have at least one copy of 132L. LaCava et al. (2021) reported an increase in frequency of 225F—an allele associated with slowed CWD progression—in MD in areas of high disease prevalence and further showed that the 225F allele was detected in herds with a longer known history of CWD prevalence. Over a 17‐year period, Pilot et al. (2025) similarly detected a significant increase in the G96S allele in farmed herds of WTD in Alberta and Saskatchewan. While likely advantageous to populations, the genetic response and inhibitory nature to disease of these alleles suggest an ongoing evolutionary arms race between host and pathogen, such that the CWD agent could in time adapt during replication to more easily infect individuals having a more resistant genotype (Race et al. 2011).

Aside from locus G96S, we did not detect additional relationships between CWD status and polymorphisms in WTD *PRNP*, including those previously described [e.g., A116G (Haley et al. 2019; O'Rourke et al. 2004)]. For some loci, where the polymorphism was present (A116G), it was not frequent enough in the population to assess the relationship with CWD status.

Overall, this study contributes to the growing body of evidence that the 96S allele of the *PRNP* gene confers resilience to CWD. The tendency for individuals with at least one copy of 96S to be CWD negative was clear, and we found some evidence of response to selection in regions with a longer history of CWD. With increasing CWD incidence and the potential for evolution in response to CWD selective pressures (Robinson et al. 2012; Hoar et al. 2024), we predict a further shift in allele frequencies over time in response to known CWD hotspots.

## Author Contributions

**Christine M. Bubac:** investigation (equal), methodology (equal), writing – original draft (equal), writing – review and editing (lead). **Catherine I. Cullingham:** conceptualization (equal), data curation (equal), formal analysis (equal), investigation (equal), methodology (equal), project administration (equal), writing – original draft (equal), writing – review and editing (equal). **David W. Coltman:** conceptualization (equal), investigation (equal), methodology (equal), project administration (equal), writing – review and editing (equal). **Debbie McKenzie:** conceptualization (equal), methodology (equal), project administration (equal), writing – review and editing (equal). **Ty Russell:** data curation (equal), investigation (equal), methodology (equal), writing – original draft (equal), writing – review and editing (equal). **Margo J. Pybus:** conceptualization (equal), data curation (equal), writing – review and editing (equal). **Mark C. Ball:** conceptualization (equal), data curation (equal), writing – review and editing (equal).

## Conflicts of Interest

The authors declare no conflicts of interest.

## Supporting information


**Data S1:** ece372449‐sup‐0001‐Supinfo.xlsx.

**Figures S1–S5:** ece372449‐sup‐0002‐FiguresS1‐S5.pdf.

**Table S1:** ece372449‐sup‐0003‐Supplementaltable1.docx.

## Data Availability

Data and scripts for this work can be found on Borealis: “Spatial and temporal patterns of prion gene variation are consistent with a response to chronic wasting disease induced selection in wild white‐tailed deer” https://doi.org/10.5683/SP3/NSV8OU. A map of wildlife management units with their labels is included in the supplements (Figure S1).
