# Supplementary material for: Spatial and Temporal Patterns of Prion Gene Variation Are Consistent With a Response to Chronic Wasting Disease‐Induced Selection in Wild White‐Tailed Deer
Source: Ecol Evol. 2025 Nov 14;15(11):e72449. doi: 10.1002/ece3.72449 (PMC12617257; doi:10.1002/ece3.72449)
Supplement: Supplementary file 2 — Figures S1–S5: ece372449‐sup‐0002‐FiguresS1‐S5.pdf. [file ECE3-15-e72449-s002.pdf]

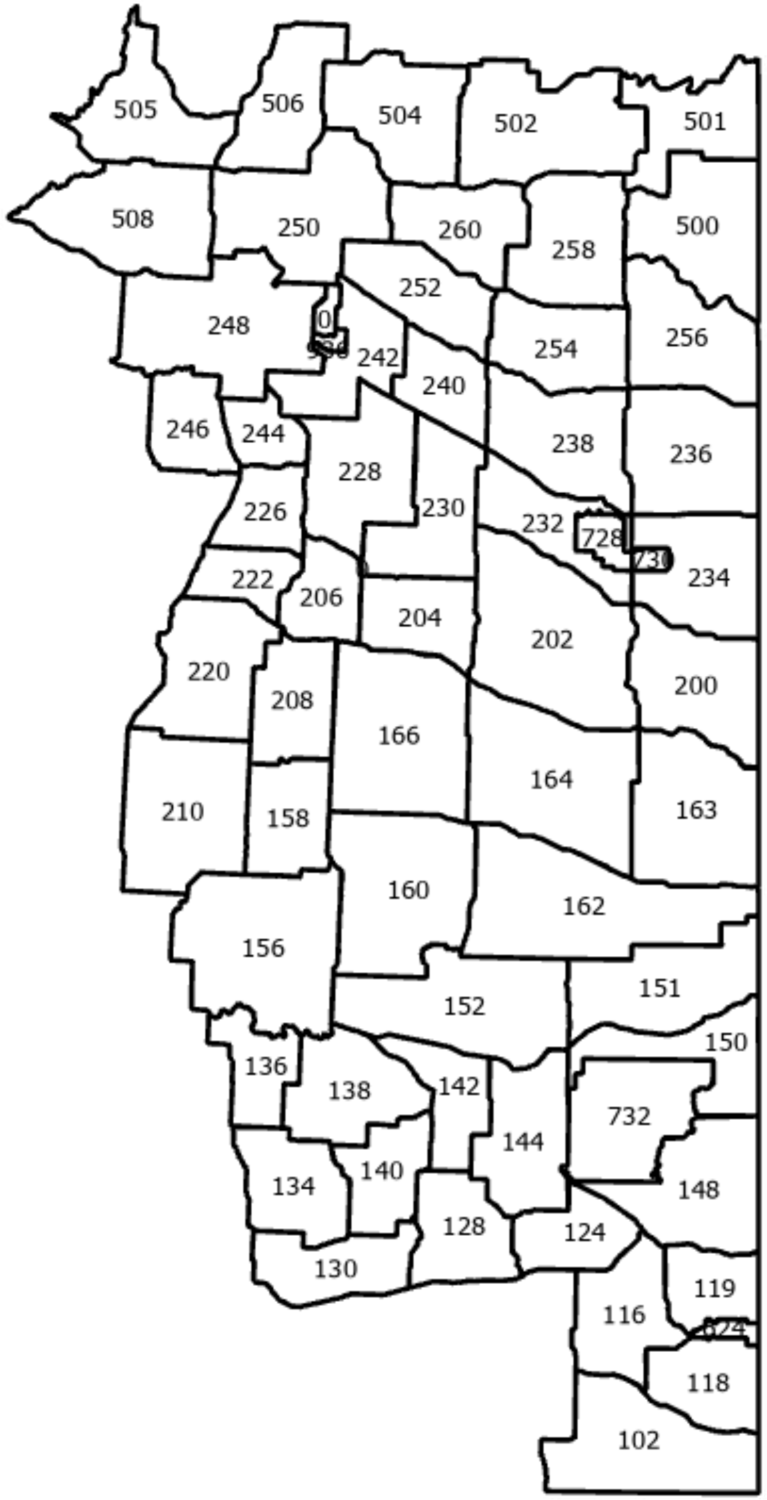

Figure S1. Map of wildlife management units in the southeastern quadrant of Alberta including unit labels

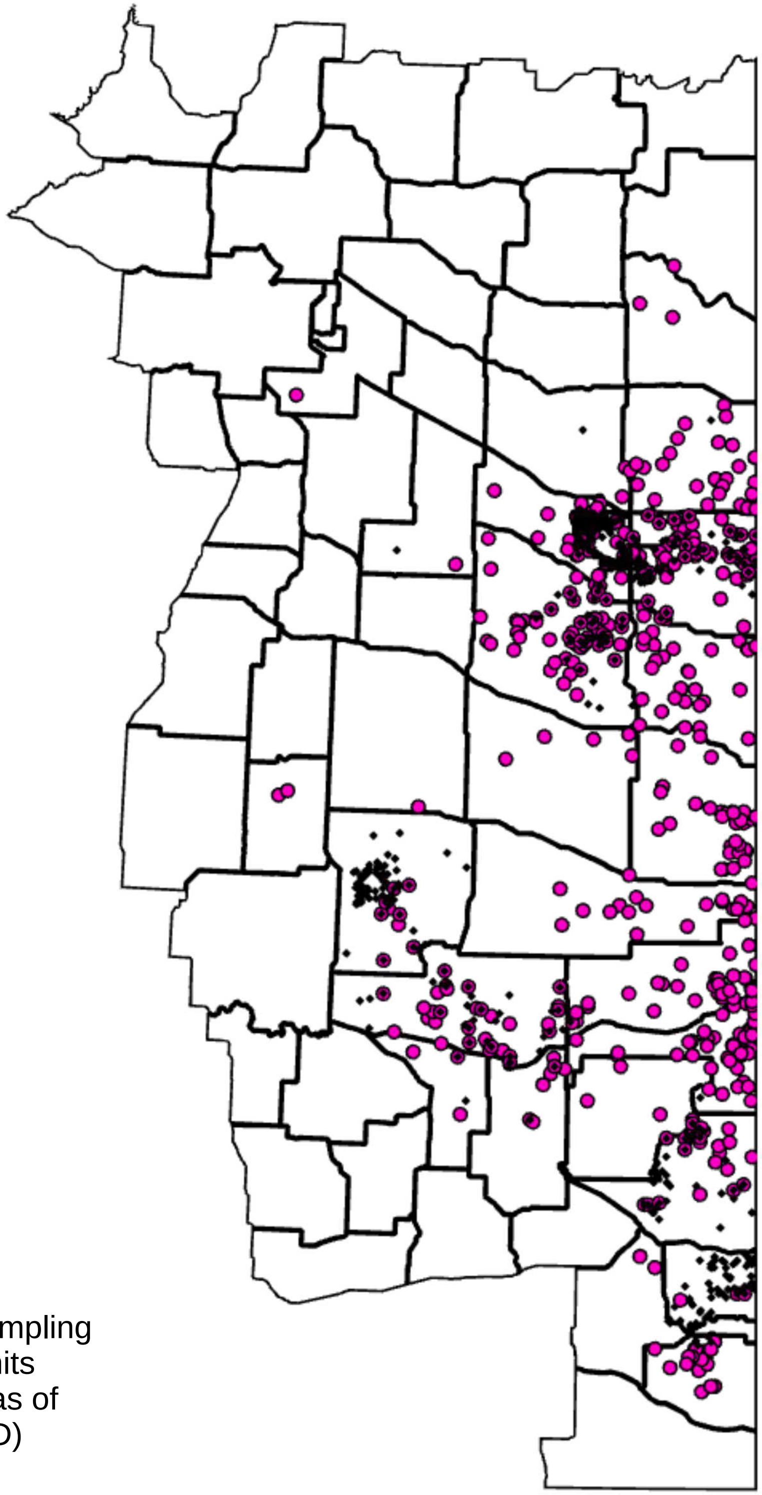

Figure S2. Mule deer (MD) sampling within wildlife management units (WMUs; 2014-2017), as well as of chronic wasting disease (CWD) occurrence in MD, in Alberta (Canada) from 2007-2016.

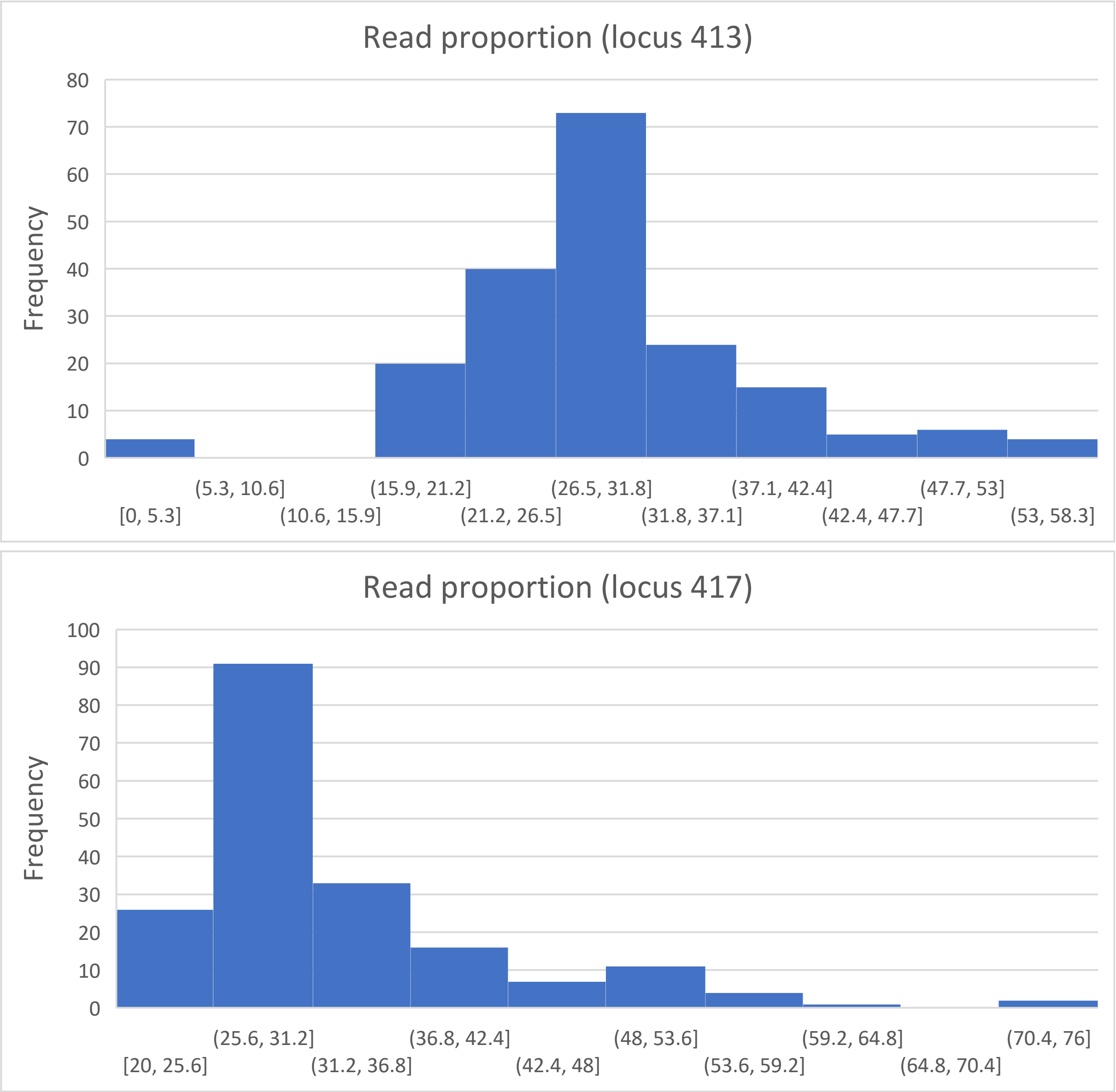

Figure S3. Histograph of the proportion of reads associated with the minor allele at locus 413 and 417 in mule deer. The expectation for a heterozygote, is that the proportion should be ~50%, however, if the locus is associated with a pseudogene and is heterozygous in one region, and homozygous in the other region, the expectation is that the proportion of reads for the minor allele would be ~25%.

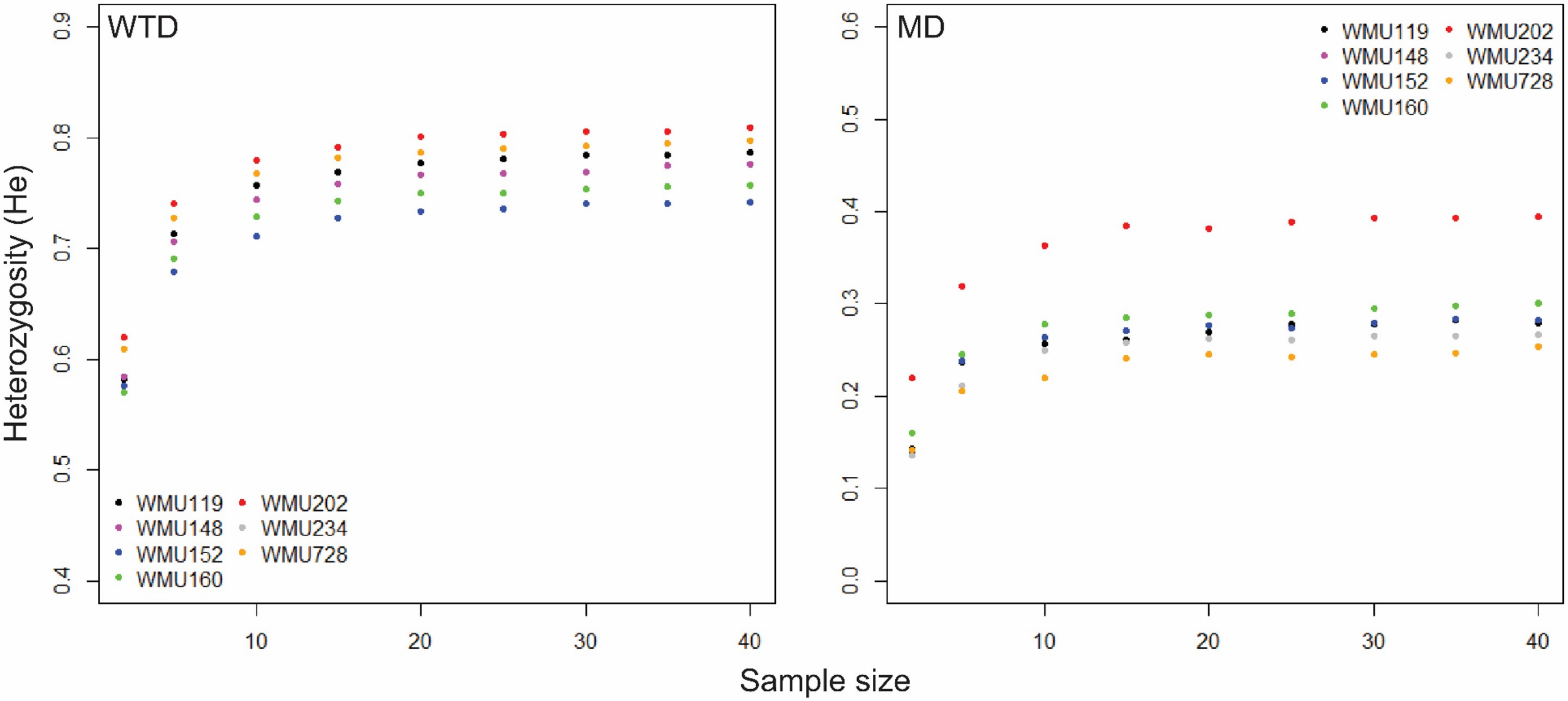

Figure S4. Rarefaction curves showing expected heterozygosity (H_E) as a function of sample size per wildlife management unit (WMU) for white-tailed deer (WTD) and mule deer (MD) in Alberta, Canada for seven WMUs. Plateaus in the curves indicate sufficient sampling occurred to capture genetic diversity for analyses.

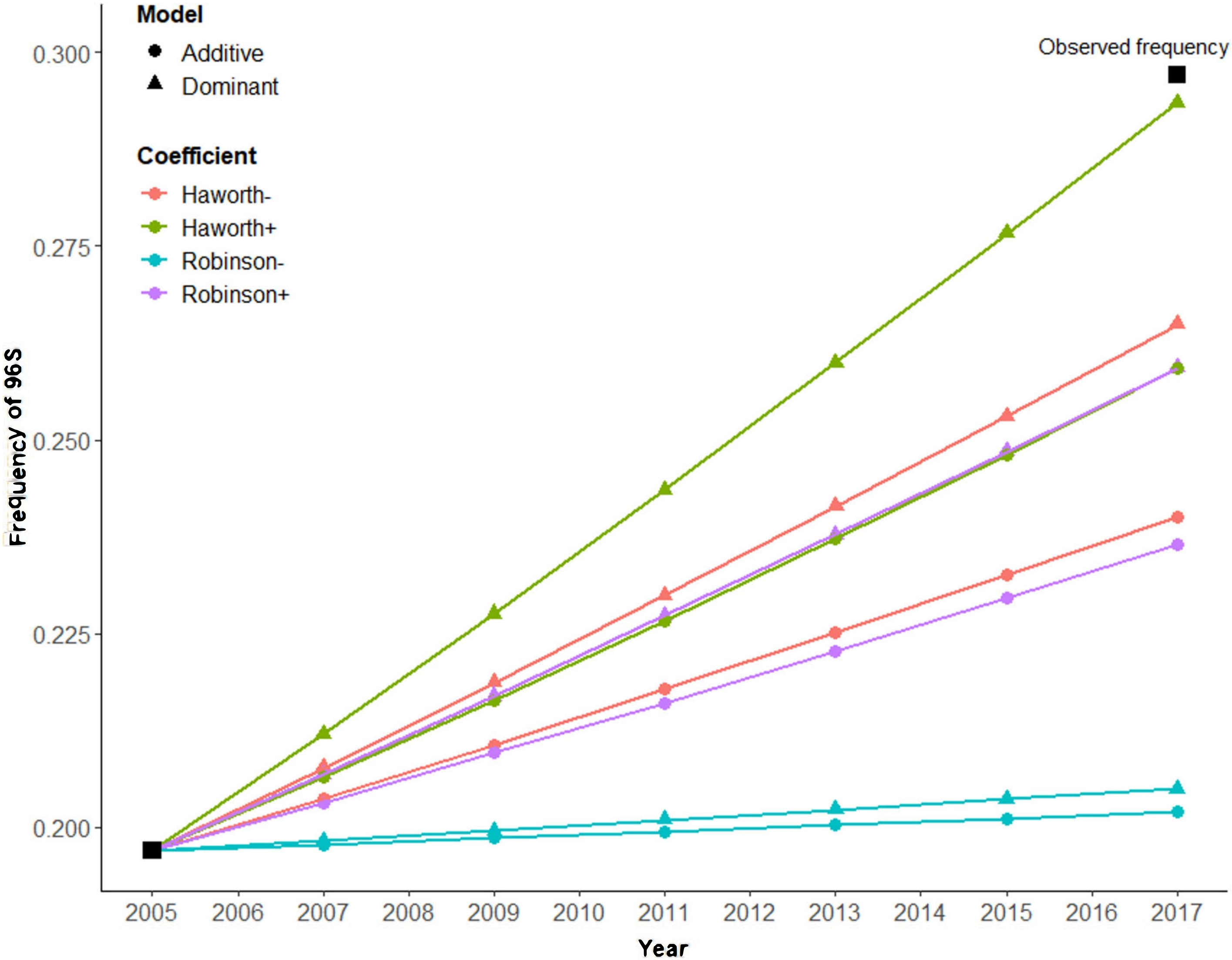

Figure S5. Predicted change over time in the allele frequency for 96S in white-tail deer based on two models of gene action ("Additive" and "Dominant"), and four estimates of selection coefficients calculated in Haworth et al. (2021), and Robinson et al. (2012). The negative "-" and plus **"+"** signs denote the upper and lower estimates, respectively. Observed values are denoted by black squares - the starting allele frequency for this allele in WMU 151 was obtained from Wilson et al. (2009) (N = 33), and the value for 2017 was estimated from 37 individuals we sampled in 2017.
