# Supplementary material for: Spatial and Temporal Patterns of Prion Gene Variation Are Consistent With a Response to Chronic Wasting Disease‐Induced Selection in Wild White‐Tailed Deer
Source: Ecol Evol. 2025 Nov 14;15(11):e72449. doi: 10.1002/ece3.72449 (PMC12617257; doi:10.1002/ece3.72449)
Supplement: Supplementary file 3 — Table S1: ece372449‐sup‐0003‐Supplementaltable1.docx. [file ECE3-15-e72449-s003.docx]

**Supplemental Information for:**

**Spatial and temporal patterns of prion gene variation are consistent with a response to chronic wasting disease induced selection in wild white-tailed deer**

Bubac CM, Russel T, McKenzie D, Ball MC, Pybus MJ, Coltman DW, Cullingham CI

**Supplemental Table S1.** Parameter estimates of additive and dominant genetic models generated from generalized linear model analyses assessing sources of variation (genotype and sex) on CWD status in white-tailed deer in Alberta, Canada. The following were true for both models: null deviance = 609.11 with df = 2130; residual deviance = 554.14 (additive) and 554.59 (dominant) with df = 2128; and AIC = 560.14 (additive) and 560.59 (dominant).

|  | **Additive Genetic Model** | | | | **Dominant Genetic Model** | | | |
| --- | --- | --- | --- | --- | --- | --- | --- | --- |
| **Coefficients** | **Estimate** | **SE** | **z** | ***p* value** | **Estimate** | **SE** | **z** | ***p* value** |
| **(Intercept)** | -5.794 | 0.6875 | -8.428 | <0.0005 | -5.7805 | 0.6875 | -8.408 | <0.0005 |
| **Genotype** | -1.215 | 0.2806 | -4.329 | <0.0005 | -0.6749 | 0.1519 | -4.443 | <0.0005 |
| **Sex (M)** | 1.6437 | 0.3611 | 4.552 | <0.0005 | 1.64 | 0.3611 | 4.541 | <0.0005 |
